# Supplementary material for: The Dysregulation of Tuning Receptors and Transcription Factors in the Antennae of Orco and Ir8a Mutants in Aedes aegypti Suggests a Chemoreceptor Regulatory Mechanism Involving the MMB/dREAM Complex
Source: Insects. 2025 Jun 17;16(6):638. doi: 10.3390/insects16060638 (PMC12193925; doi:10.3390/insects16060638)
Supplement: Supplementary file 1 [file insects-16-00638-s001.zip › Supplementary_Folder_S1/appXSTREME_5.5.71744378849113-68636999/fimo_out_1/fimo.html]

FIMO Results


---

|  |  |  |  |  |  |
| --- | --- | --- | --- | --- | --- |
| **Database and Motifs** | **High-scoring Motif Occurences** | **Debugging Information** | **Results in TSV Format** | **Results in GFF3 Format** | **Best Site per Sequence** |

  
  


---
