# Supplementary material for: The Dysregulation of Tuning Receptors and Transcription Factors in the Antennae of Orco and Ir8a Mutants in Aedes aegypti Suggests a Chemoreceptor Regulatory Mechanism Involving the MMB/dREAM Complex
Source: Insects. 2025 Jun 17;16(6):638. doi: 10.3390/insects16060638 (PMC12193925; doi:10.3390/insects16060638)
Supplement: Supplementary file 1 [file insects-16-00638-s001.zip › Supplementary_Folder_S1/appXSTREME_5.5.71744378849113-68636999/sea_disc_out/sea.html]

SEA results


[close ]

[close ]

[
close ]

[
close ]

[
close ]

[
close ]

[
close ]

[
close ]

[
close ]

[
close ]

[
close ]

[
close ]

[
close ]

[
close ]

[
close ]

[
close ]

[
close ]

# SEA

## Simple Enrichment Analysis

For further information on how to interpret these results please access
https://meme-suite.org/meme/doc/sea-output-format.html.  
To get a copy of the MEME software please access
https://meme-suite.org.

Enriched Motifs
  |  
Input Files
  |  
Program information
  |  
Results in TSV Format 

|  
Matching Sequences

|  
Matching Sites


# Javascript is required to view these results!


## Enriched Motifs

| Logo | Database | ID | Alt ID | *P*-value | *E*-value | Q-value | TP | FP | Enrichment Ratio | Score Threshold |
| --- | --- | --- | --- | --- | --- | --- | --- | --- | --- | --- |

## Input Files

#### Alphabet


#### Sequences

#### Motifs

| Database | Source | Motif Count |
| --- | --- | --- |

#### Other Settings

|  |  |
| --- | --- |
| Strand Handling | This alphabet only has one strand. Only the given strand is processed. Both the given and reverse complement strands are processed. |
| Objective Function |  |
| Statistical Test |  |
| Sequence Shuffling |  |
| Hold-out Set |  |
| Pseudocount |  |
| Significance threshold |  |
| Random Number Seed |  |
| Trimming of Control Sequences |  |

##### SEA version

(Release date: )

##### Command line summary
