# Supplementary material for: The Dysregulation of Tuning Receptors and Transcription Factors in the Antennae of Orco and Ir8a Mutants in Aedes aegypti Suggests a Chemoreceptor Regulatory Mechanism Involving the MMB/dREAM Complex
Source: Insects. 2025 Jun 17;16(6):638. doi: 10.3390/insects16060638 (PMC12193925; doi:10.3390/insects16060638)
Supplement: Supplementary file 1 [file insects-16-00638-s001.zip › Supplementary_Folder_S1/appXSTREME_5.5.71744378849113-68636999/xstreme.html]

XSTREME Results


[close ]

[close ]

[
close ]

[
close ]

[
close ]

[
close ]

[
close ]

[
close ]

[
close ]

[
close ]

[
close ]

[
close ]

# XSTREME

## Motif Discovery and Enrichment Analysis

For further information on how to interpret these results please access
https://meme-suite.org/meme/doc/xstreme-output-format.html.  
To get a copy of the MEME software please access
https://meme-suite.org.

Motifs
  |  
Programs
  |  
Input Files
  |  
Program information
  |  
Summary in TSV Format 
  |  
Non-redundant Motifs in MEME Text Format


# Javascript is required to view these results!

# Your browser does not support canvas!


## Motifs

**Enriched motifs
(E-value ≤ ).**

Expand All Clusters
Collapse All Clusters

## Programs

## Input Files

#### Alphabet

#### Motifs

##### XSTREME version

(Release date: )

##### Command line
