# Supplementary figures and images for: The Dysregulation of Tuning Receptors and Transcription Factors in the Antennae of Orco and Ir8a Mutants in Aedes aegypti Suggests a Chemoreceptor Regulatory Mechanism Involving the MMB/dREAM Complex
Source: Insects. 2025 Jun 17;16(6):638. doi: 10.3390/insects16060638 (PMC12193925; doi:10.3390/insects16060638)

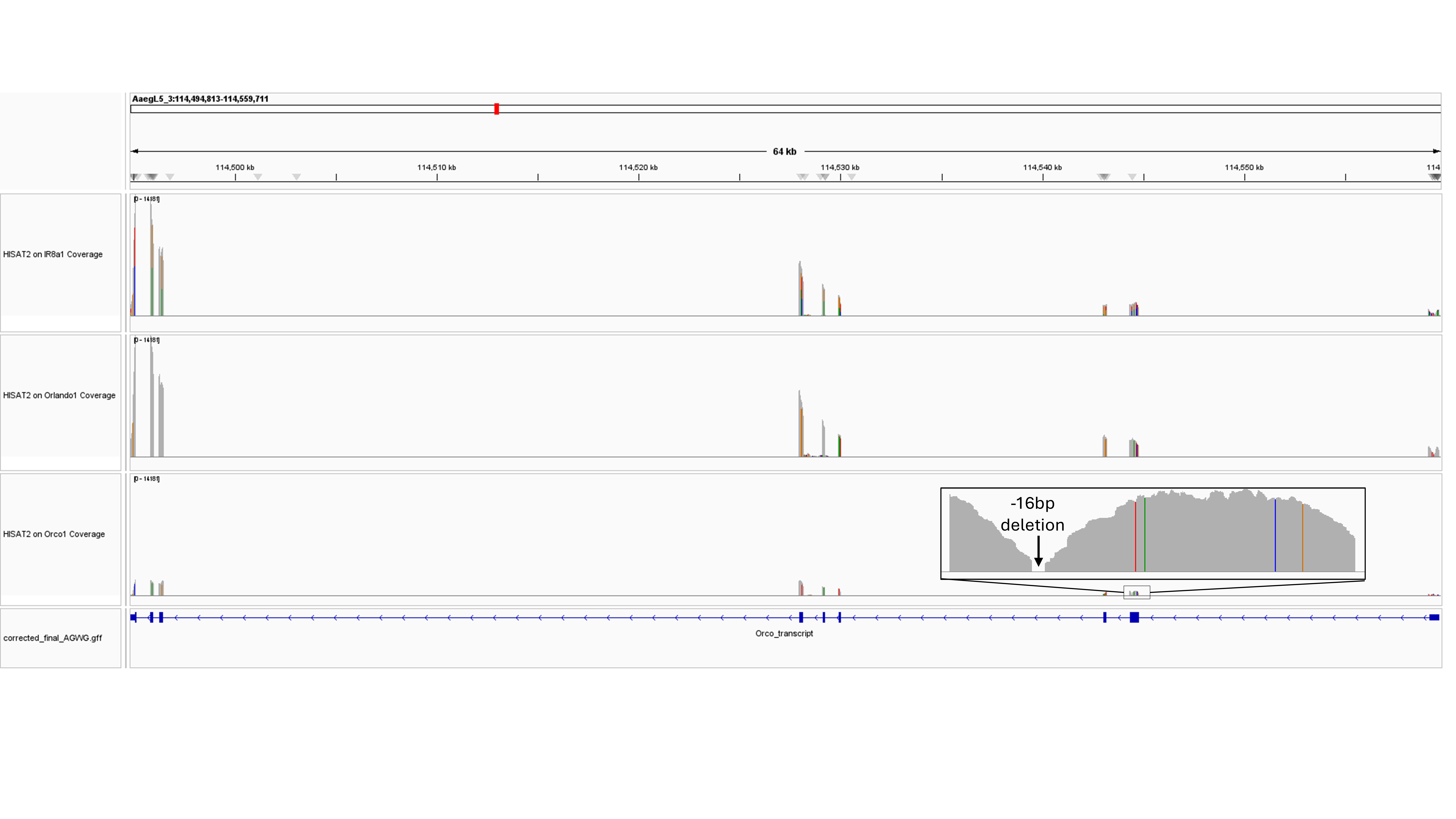

Supplement: Supplementary file 1 [file insects-16-00638-s001.zip › Figure_S1.PNG]

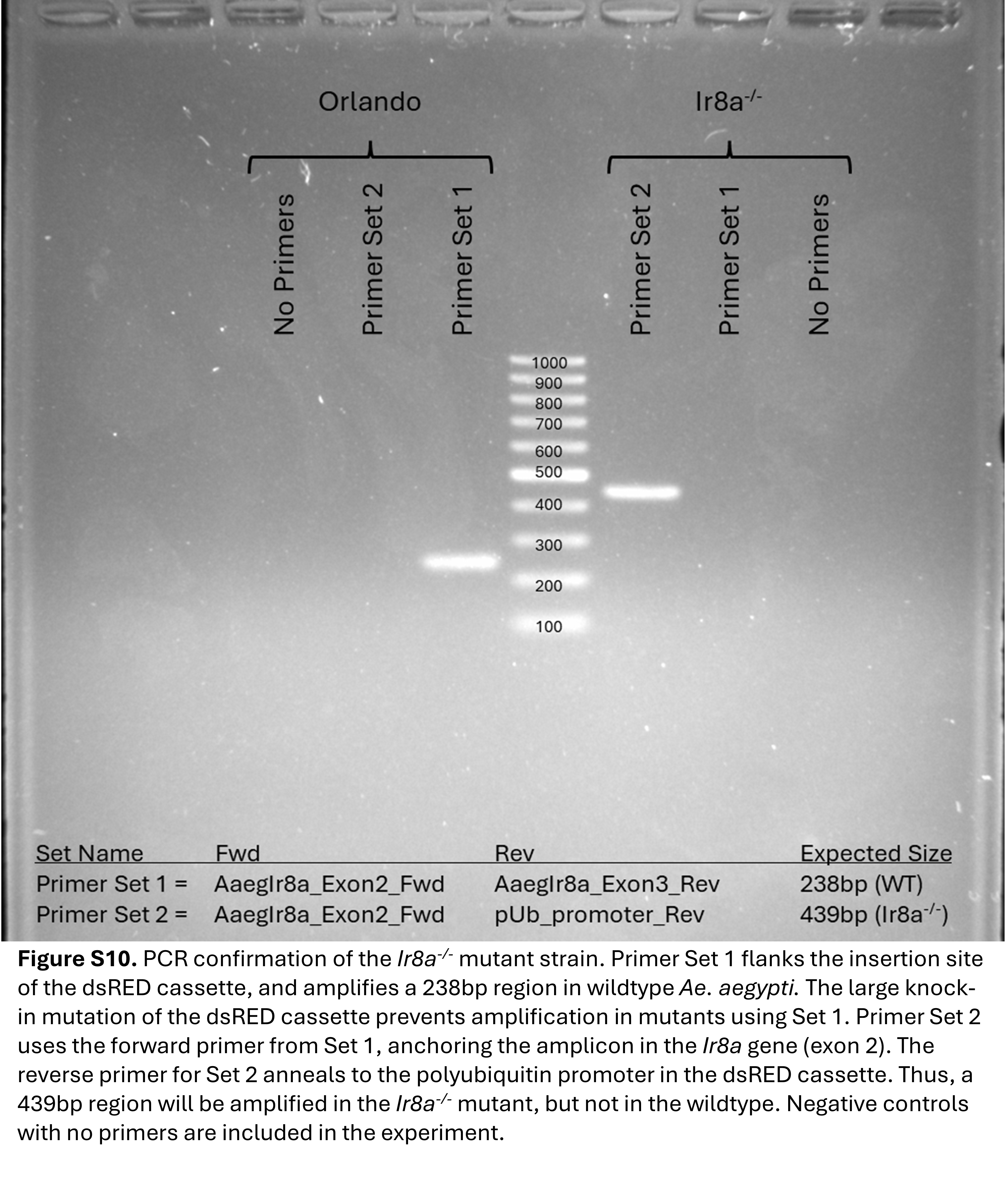

Supplement: Supplementary file 1 [file insects-16-00638-s001.zip › Figure_S10.tiff]

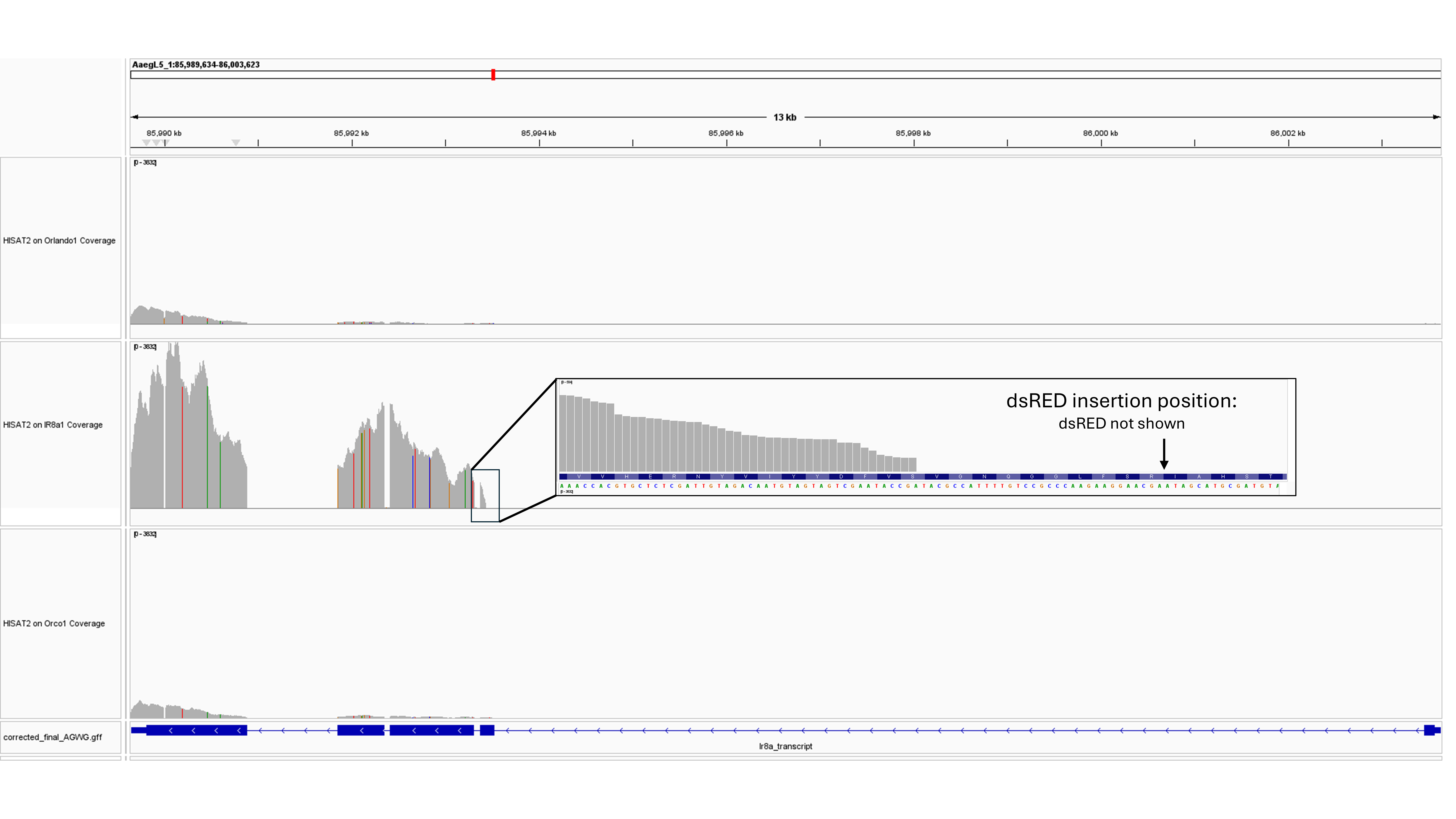

Supplement: Supplementary file 1 [file insects-16-00638-s001.zip › Figure_S2.PNG]

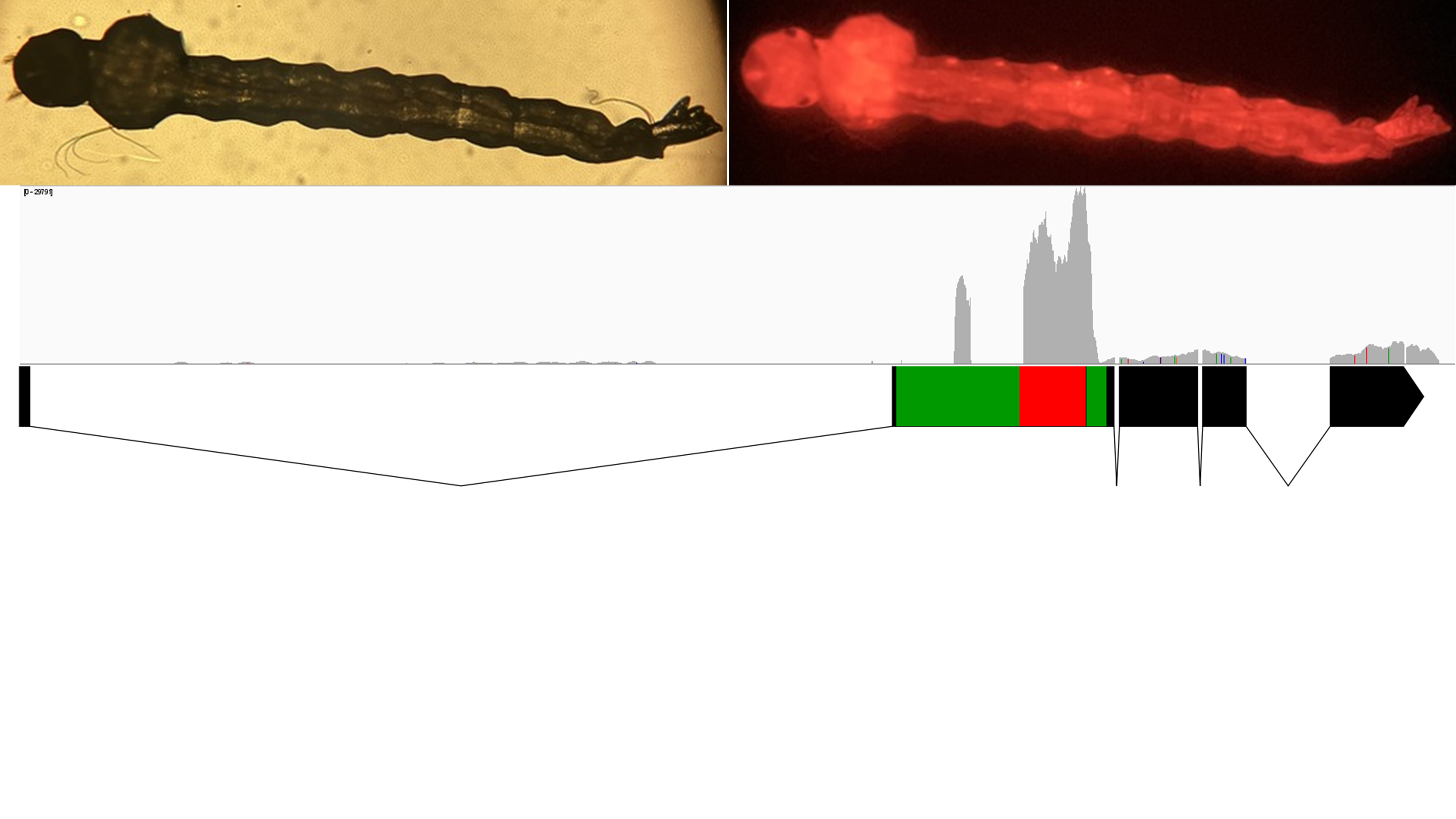

Supplement: Supplementary file 1 [file insects-16-00638-s001.zip › Figure_S3.png]

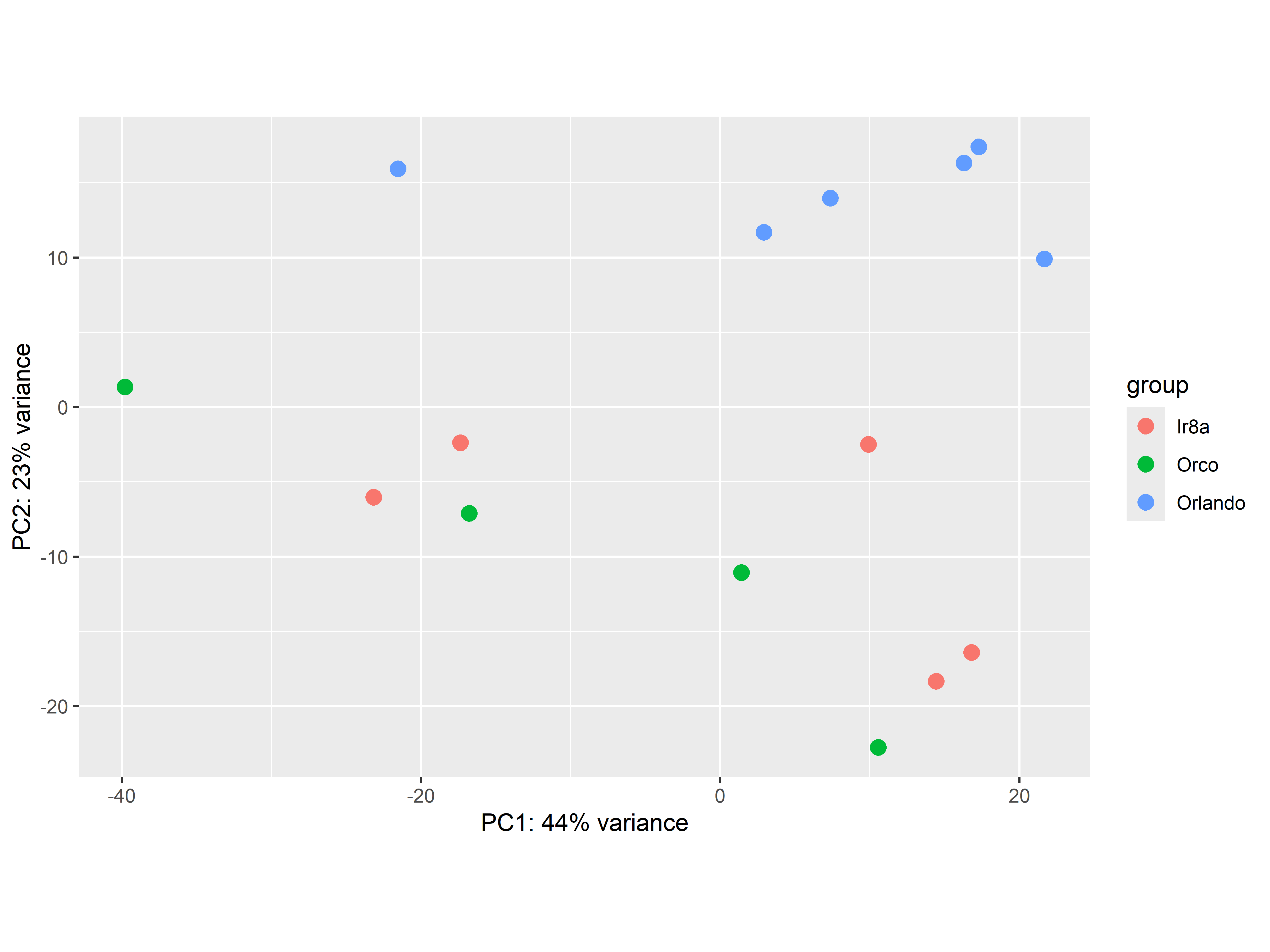

Supplement: Supplementary file 1 [file insects-16-00638-s001.zip › Figure_S4.png]

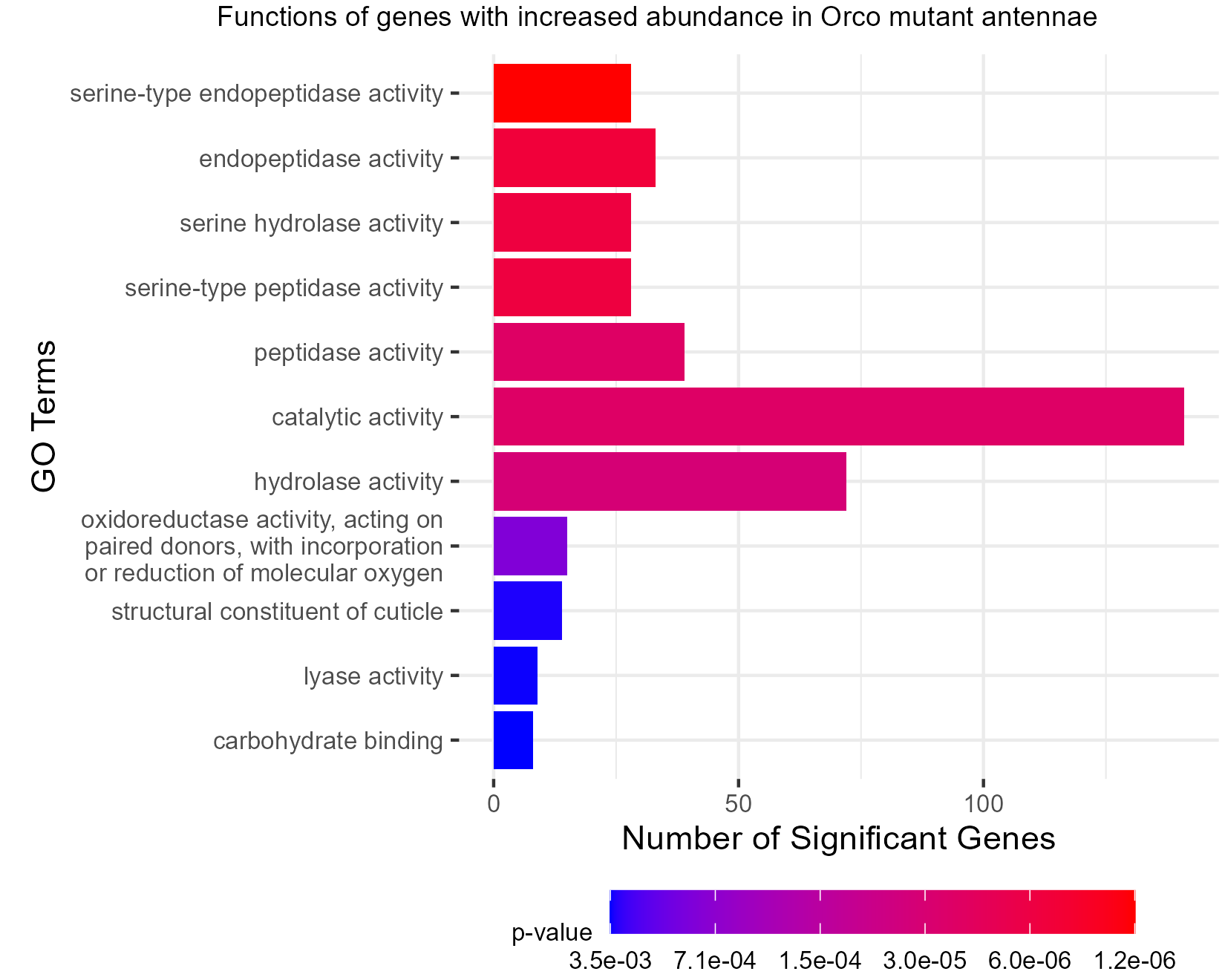

Supplement: Supplementary file 1 [file insects-16-00638-s001.zip › Figure_S5.png]

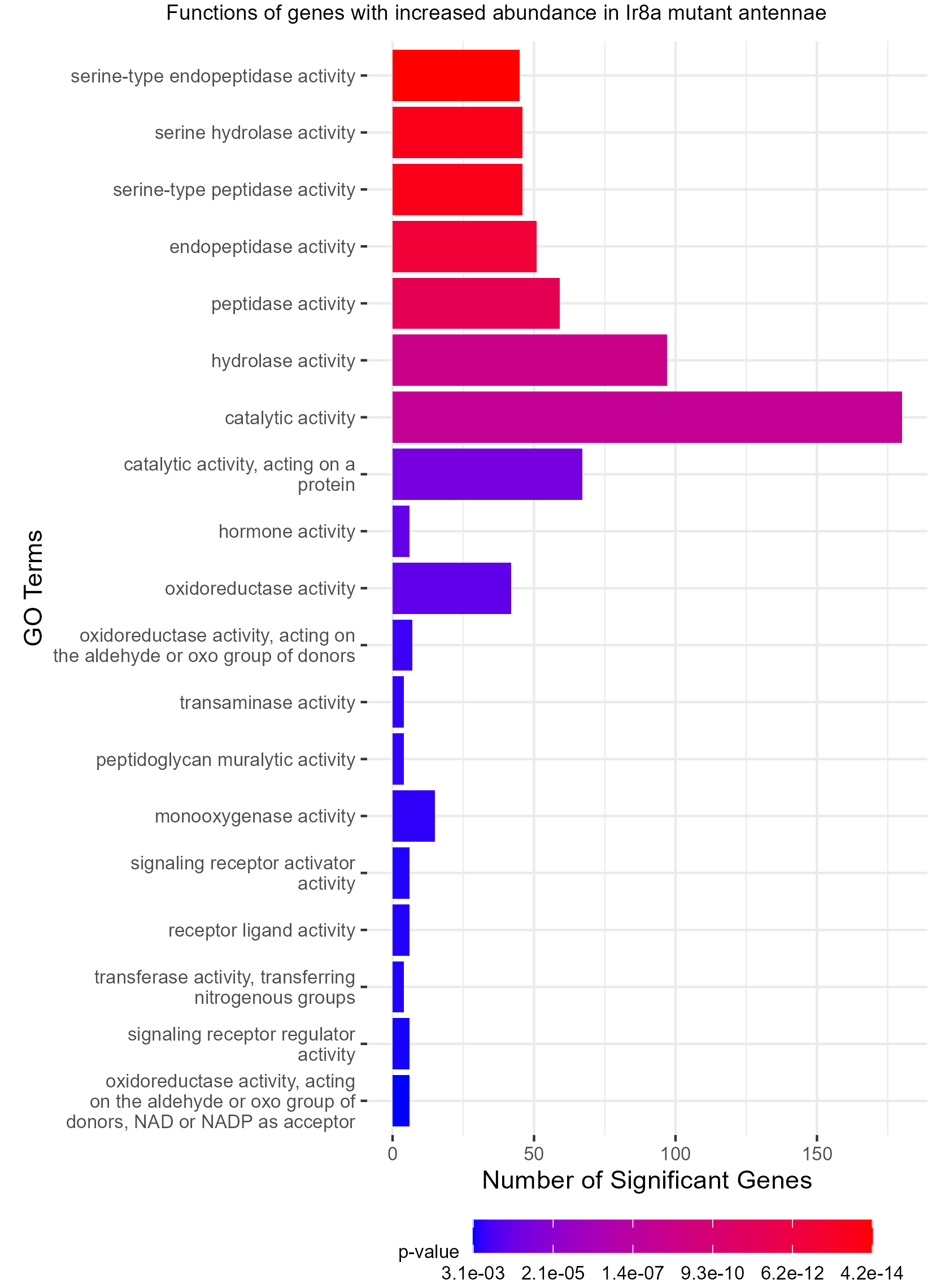

Supplement: Supplementary file 1 [file insects-16-00638-s001.zip › Figure_S6.png]

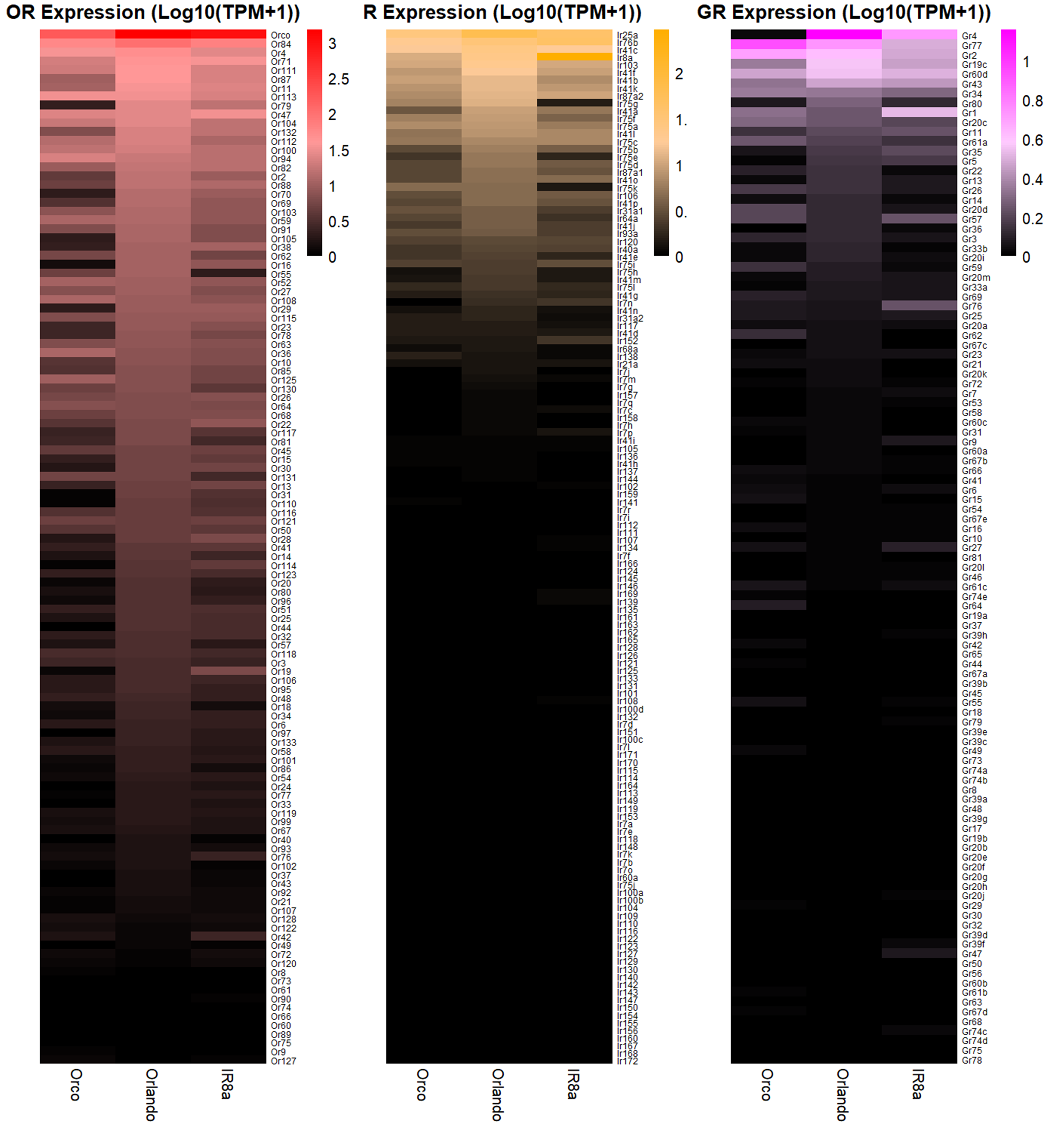

Supplement: Supplementary file 1 [file insects-16-00638-s001.zip › Figure_S7.PNG]

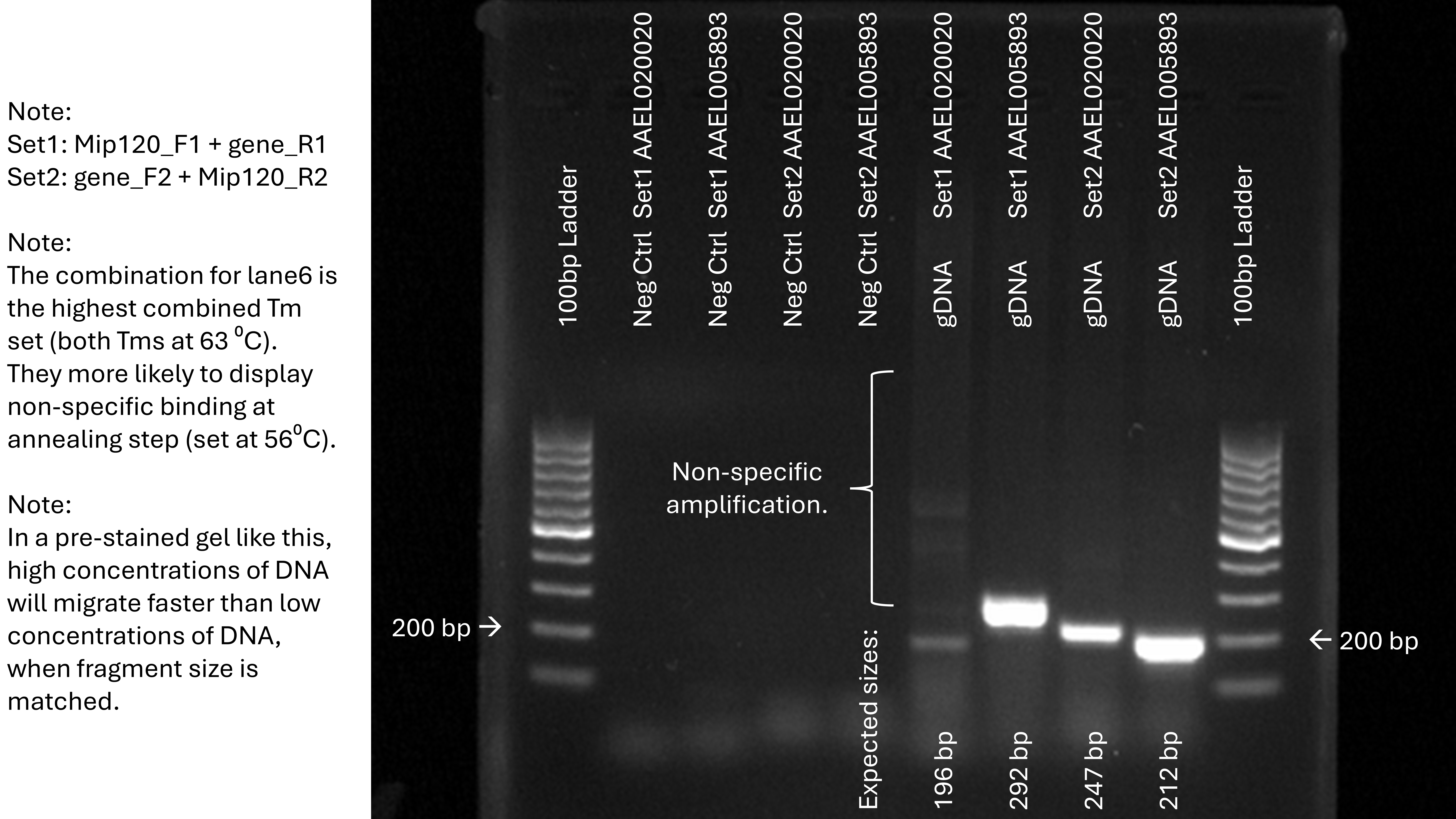

Supplement: Supplementary file 1 [file insects-16-00638-s001.zip › Figure_S8.png]

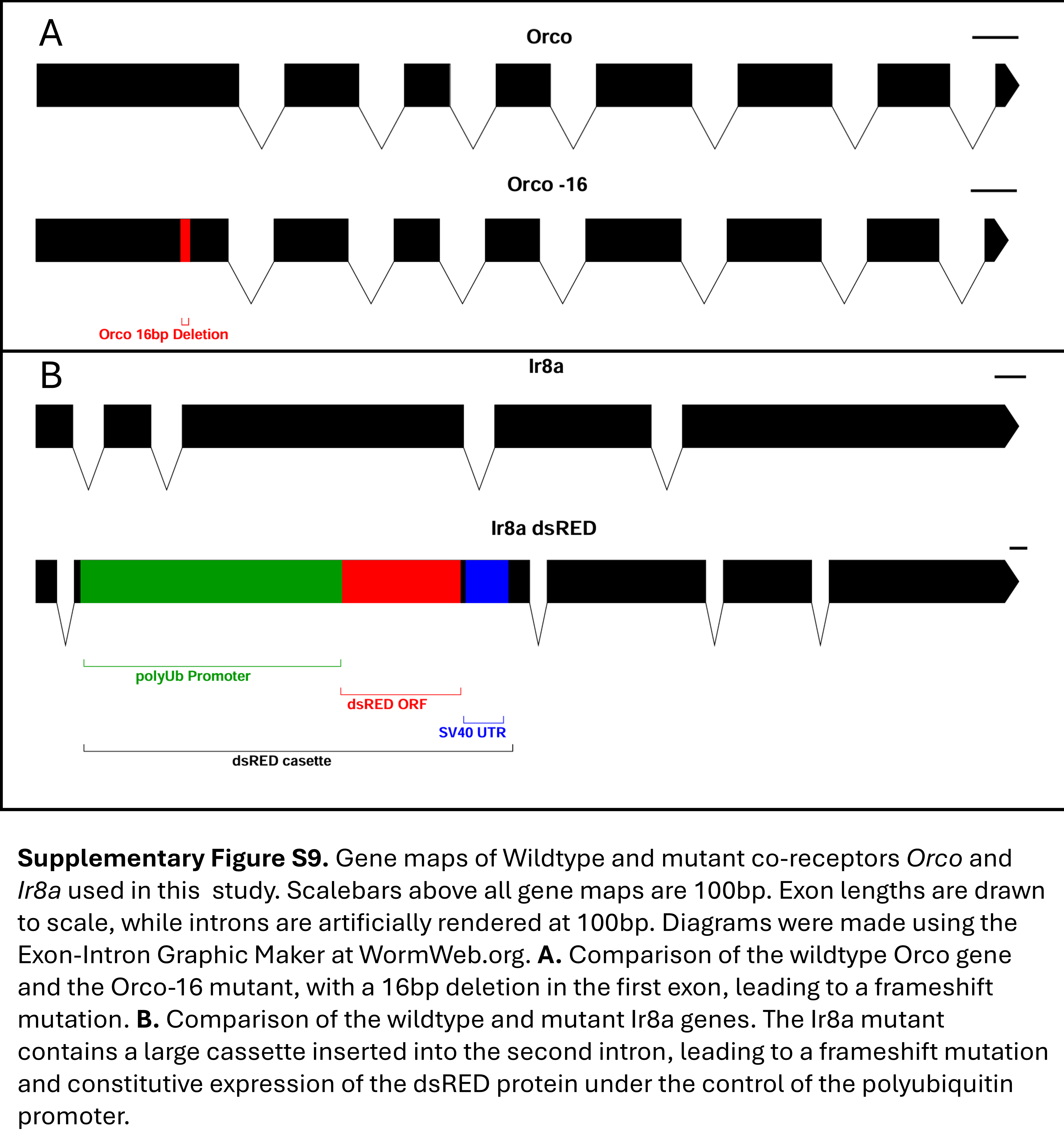

Supplement: Supplementary file 1 [file insects-16-00638-s001.zip › Figure_S9.tiff]

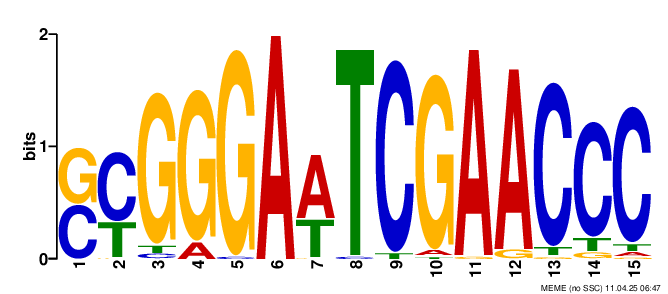

Supplement: Supplementary file 1 [file insects-16-00638-s001.zip › Supplementary_Folder_S1/appXSTREME_5.5.71744378849113-68636999/meme_out/logo1.png]

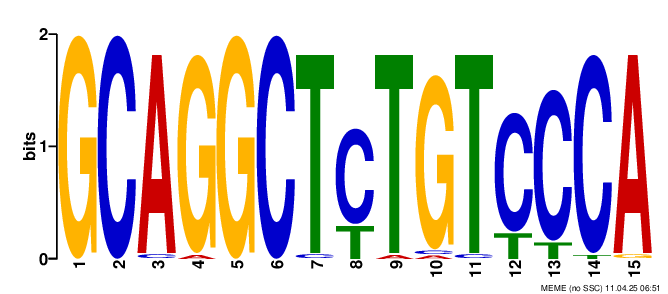

Supplement: Supplementary file 1 [file insects-16-00638-s001.zip › Supplementary_Folder_S1/appXSTREME_5.5.71744378849113-68636999/meme_out/logo2.png]

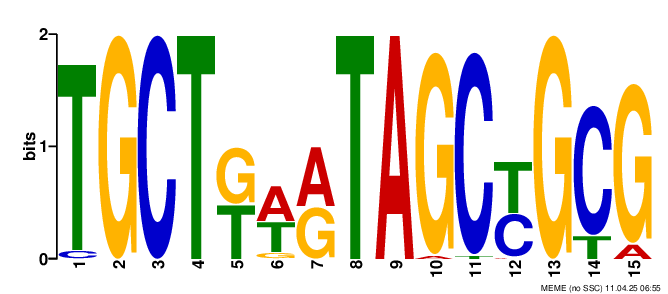

Supplement: Supplementary file 1 [file insects-16-00638-s001.zip › Supplementary_Folder_S1/appXSTREME_5.5.71744378849113-68636999/meme_out/logo3.png]

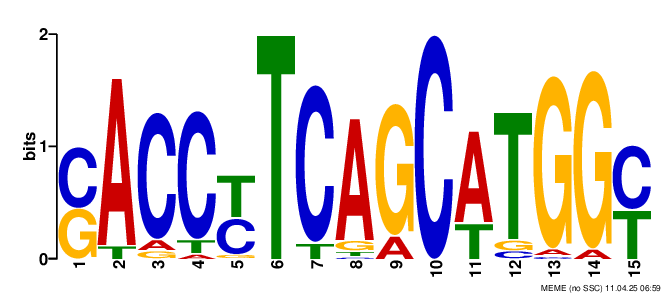

Supplement: Supplementary file 1 [file insects-16-00638-s001.zip › Supplementary_Folder_S1/appXSTREME_5.5.71744378849113-68636999/meme_out/logo4.png]

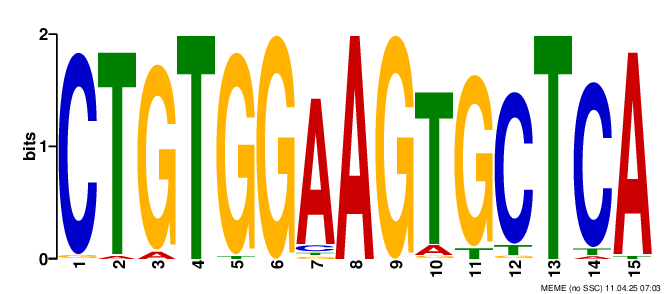

Supplement: Supplementary file 1 [file insects-16-00638-s001.zip › Supplementary_Folder_S1/appXSTREME_5.5.71744378849113-68636999/meme_out/logo5.png]

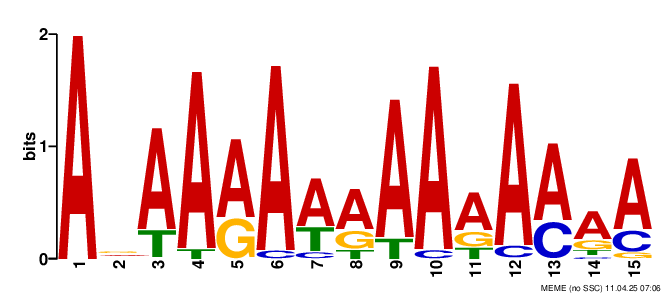

Supplement: Supplementary file 1 [file insects-16-00638-s001.zip › Supplementary_Folder_S1/appXSTREME_5.5.71744378849113-68636999/meme_out/logo6.png]

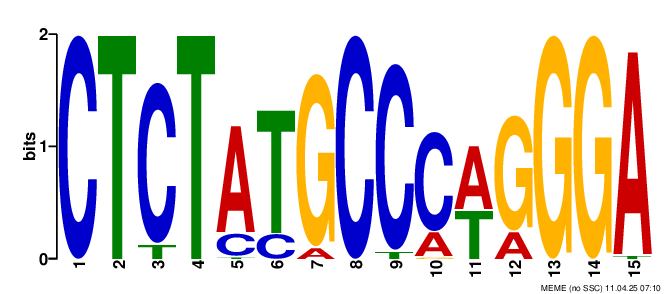

Supplement: Supplementary file 1 [file insects-16-00638-s001.zip › Supplementary_Folder_S1/appXSTREME_5.5.71744378849113-68636999/meme_out/logo7.png]

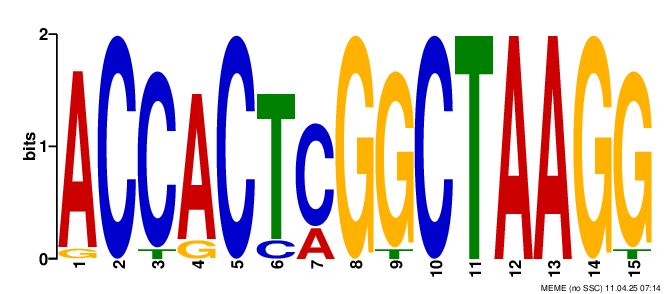

Supplement: Supplementary file 1 [file insects-16-00638-s001.zip › Supplementary_Folder_S1/appXSTREME_5.5.71744378849113-68636999/meme_out/logo8.png]

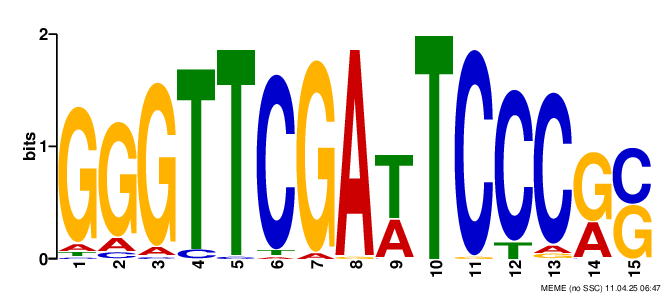

Supplement: Supplementary file 1 [file insects-16-00638-s001.zip › Supplementary_Folder_S1/appXSTREME_5.5.71744378849113-68636999/meme_out/logo_rc1.png]

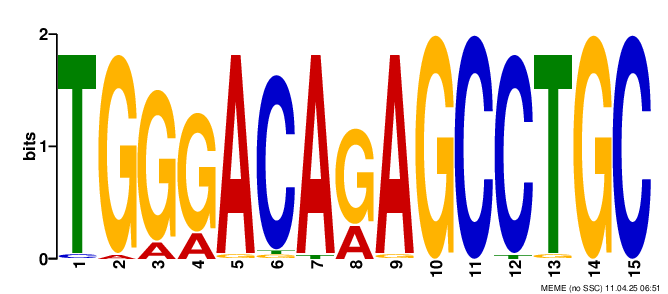

Supplement: Supplementary file 1 [file insects-16-00638-s001.zip › Supplementary_Folder_S1/appXSTREME_5.5.71744378849113-68636999/meme_out/logo_rc2.png]

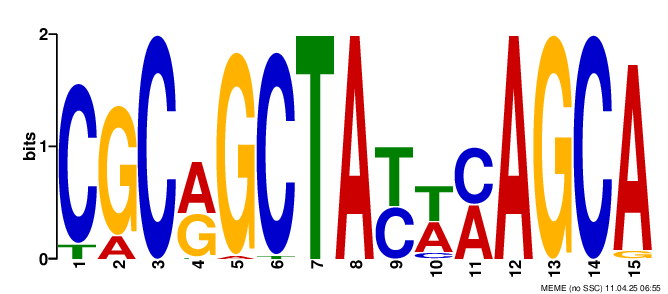

Supplement: Supplementary file 1 [file insects-16-00638-s001.zip › Supplementary_Folder_S1/appXSTREME_5.5.71744378849113-68636999/meme_out/logo_rc3.png]

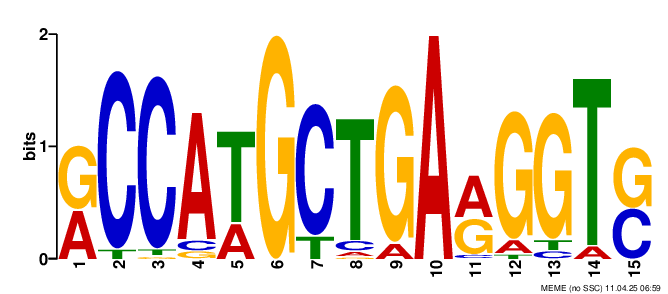

Supplement: Supplementary file 1 [file insects-16-00638-s001.zip › Supplementary_Folder_S1/appXSTREME_5.5.71744378849113-68636999/meme_out/logo_rc4.png]

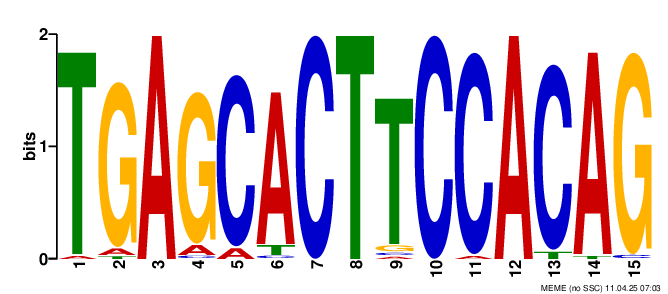

Supplement: Supplementary file 1 [file insects-16-00638-s001.zip › Supplementary_Folder_S1/appXSTREME_5.5.71744378849113-68636999/meme_out/logo_rc5.png]

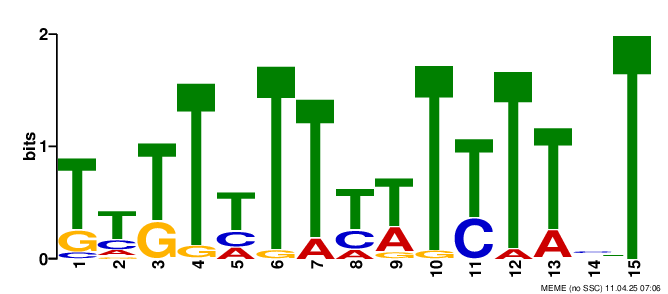

Supplement: Supplementary file 1 [file insects-16-00638-s001.zip › Supplementary_Folder_S1/appXSTREME_5.5.71744378849113-68636999/meme_out/logo_rc6.png]

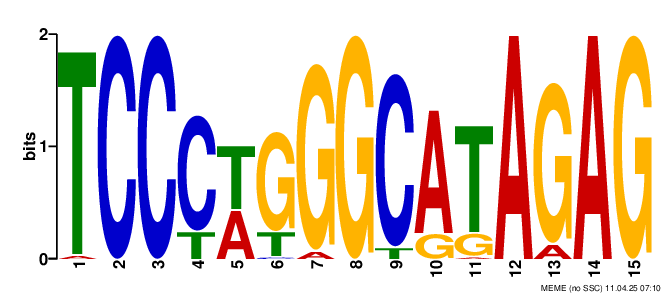

Supplement: Supplementary file 1 [file insects-16-00638-s001.zip › Supplementary_Folder_S1/appXSTREME_5.5.71744378849113-68636999/meme_out/logo_rc7.png]

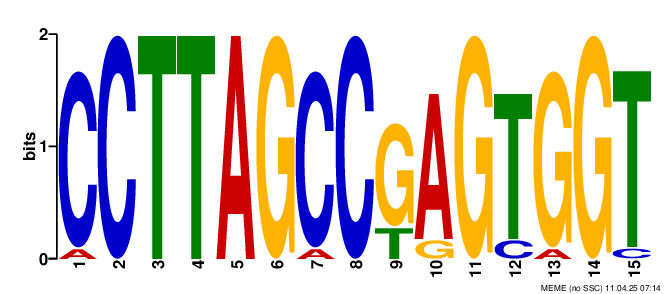

Supplement: Supplementary file 1 [file insects-16-00638-s001.zip › Supplementary_Folder_S1/appXSTREME_5.5.71744378849113-68636999/meme_out/logo_rc8.png]

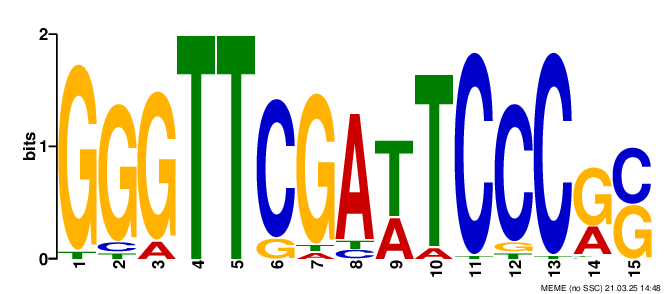

Supplement: Supplementary file 1 [file insects-16-00638-s001.zip › Supplementary_Folder_S2/appXSTREME_5.5.717425933931101286170038/meme_out/logo1.png]

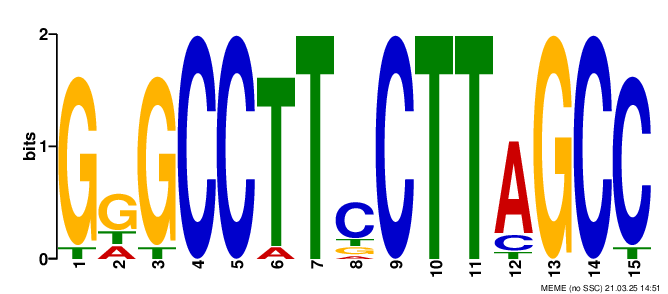

Supplement: Supplementary file 1 [file insects-16-00638-s001.zip › Supplementary_Folder_S2/appXSTREME_5.5.717425933931101286170038/meme_out/logo2.png]

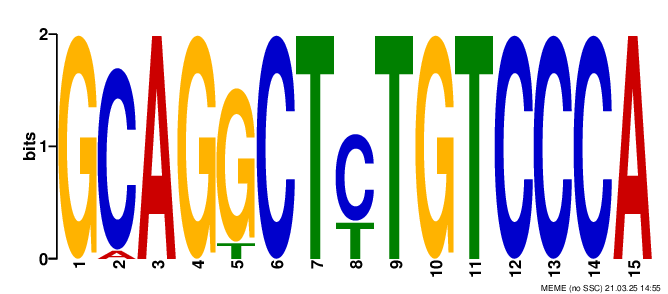

Supplement: Supplementary file 1 [file insects-16-00638-s001.zip › Supplementary_Folder_S2/appXSTREME_5.5.717425933931101286170038/meme_out/logo3.png]

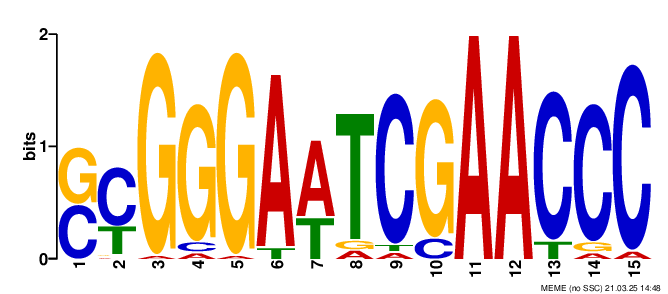

Supplement: Supplementary file 1 [file insects-16-00638-s001.zip › Supplementary_Folder_S2/appXSTREME_5.5.717425933931101286170038/meme_out/logo_rc1.png]

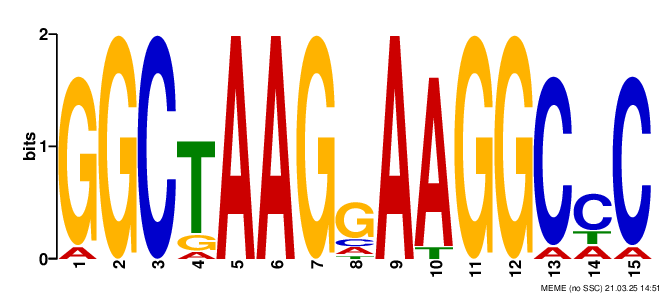

Supplement: Supplementary file 1 [file insects-16-00638-s001.zip › Supplementary_Folder_S2/appXSTREME_5.5.717425933931101286170038/meme_out/logo_rc2.png]

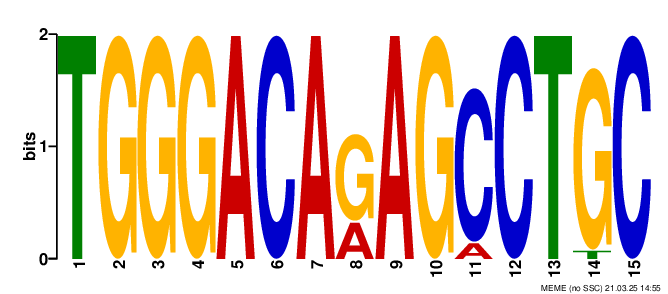

Supplement: Supplementary file 1 [file insects-16-00638-s001.zip › Supplementary_Folder_S2/appXSTREME_5.5.717425933931101286170038/meme_out/logo_rc3.png]
